# Exploring the intentions of pharmacy students towards pharmacy ownership by using theory of planned behaviour

**DOI:** 10.1186/s13104-016-1996-4

**Published:** 2016-03-22

**Authors:** Muhammad Umair Khan, Akram Ahmad, Muhammad Fayyaz, Nida Ashraf, Akshaya Bhagavathula

**Affiliations:** Department of Clinical Pharmacy, UCSI University, Cheras, 56000 Kuala Lumpur, Malaysia; Department of Pharmaceutics, Faculty of Pharmacy, Hamadard University, Karachi, Pakistan; Department of Pharmaceutics, Faculty of Pharmacy, University of Karachi, Karachi, Pakistan; Department of Clinical Pharmacy Research, College of Medicine and Health Sciences, University of Gondar, Gondar, Ethiopia

**Keywords:** Entrepreneurship, Pharmacy, Students, Ownership, Pakistan

## Abstract

**Objective:**

The objective of this study was to assess the association of the constructs of theory of planned behaviour (behavioural beliefs, normative beliefs, control beliefs) and demographic variables with the intentions of pharmacy students to become pharmacy owner.

**Methods:**

A cross sectional study was conducted between October and November, 2014, using a pretested, self-administered questionnaire delivered to a sample of 350 pharmacy students at a private university of Pakistan. Behavioural beliefs, normative beliefs and control beliefs were assessed on four point Likert scale of agreement. The scores were summed and dichotomized based on an arbitrary 50 % cut-off score to assess positive and negative beliefs. Binary logistic regression was used to analyse the data.

**Results:**

A total of 313 participants (89.4 %) responded to the questionnaire. Participants’ behavioural beliefs, normative beliefs and control beliefs were negative towards pharmacy ownership with the mean scores of 13.90  ± 0.41 (score range: 6–24), 9.66 ± 0.49 (score range: 4–16) and 16.88 ± 0.40 (score range: 7–28) respectively. Professional year and family business were significantly associated with intentions of pharmacy students to own a pharmacy (p < 0.05).

**Conclusion:**

Behavioural beliefs, normative beliefs and control beliefs were negative towards pharmacy ownership. Implementation of entrepreneurship course in pharmacy school may transform the beliefs of pharmacy students towards pharmacy ownership.

## Background

Due to economic crises, the stability of jobs is no longer offers by the employers. Many factors could be related to potential unemployment including advancement of technology and the hiring of low paid employees like pharmacy technicians are the biggest threat to pharmacist [1]. Additionally, researchers doubt that moves towards relaxing industry regulation may soon allow ownership of pharmacies by non-pharmacists like supermarkets’, chain stores and other retailers [2]. This urges the pharmacists to enable themselves to take up the role of pharmacy owner. Looking at the holistic view of public health, being proactive, favouring innovations and taking business risks are the entrepreneurial styles of management [3]. Researchers have reported that pharmacy owners are more satisfied with their jobs and are less stressed as compared to pharmacist employed in pharmacies [4]. Similarly, a study reported positive attitudes of pharmacist towards pharmacy ownership [5]. Since entrepreneurship involves innovations, creativity and the ability to transform those to profitable business, it would be interesting to know students intent to own a pharmacy.

The concept of ownership in pharmacy curriculum is still in its infancy stage. It has been reported that although pharmacy students are well familiarized with the basic, clinical and social sciences, as well as the contemporary approaches of patient care, they still need extensive training to gain pharmacy ownership skills in order to face the future challenges of the profession [6]. Researchers have shown that students who have undertaken a course of entrepreneurship have a higher intent of pharmacy ownership [7]. In Pakistan, there is no evidence to support that entrepreneurship is being taught as a course in pharmacy curriculum, however, different pharmacy schools are teaching entrepreneurship as a component of courses like pharmacy management and pharmaceutical marketing. There is an acute shortage of qualified practicing pharmacist at community settings in Pakistan. A study indicated that pharmacy graduates are least interested to join community settings (5.9 %) as compared to Clinical/Hospital setting (45.1 %) and industrial setting (21.6 %) [8].

The conceptual framework of this study is based on the theory of planned behaviour. It suggests that the best predictor of behaviour is intention, which in turn, is a function of an attitude, subjective norms and control beliefs. Behavioural beliefs (attitude), normative belief (subjective norms) and control beliefs (perceived behavioural control) are the major constructs of theory of planned behaviour. It is a general rule that more positive the behavioural beliefs, normative beliefs and control beliefs will be, the higher would be person’s intention to the behaviour in question. The theory of planned behaviour has been often used by researchers to explain the entrepreneurship intentions of the participants [9, 10]. The objective of this study was to investigate the intention of pharmacy students towards pharmacy ownership by using the theory of planned behaviour.

## Methods

A descriptive, cross-sectional study was conducted among the pharmacy students in a private university in Karachi, Pakistan. A sampling frame involved all undergraduate pharmacy students enrolled in the university. The University offers 5 years Doctor of Pharmacy program with an enrolment of 75 students in each academic year. The pharmacy program was approved by Pharmacy Council of Pakistan and Higher Education Commission of Pakistan. The study was conveniently conducted for a period of 2 months from October to November 2014.

A self-administered questionnaire was used a tool to collect the data from the participants. The questionnaire was distributed to the students by one of the authors responsible for data collection. A primary draft of the questionnaire was designed by the authors after an extensive literature review [3, 5, 11, 12], after which it was sent to three pharmacy academicians for content validity. The expert opinion was given in view of making the questionnaire more simple, relevant and important. Questionnaire was then face validated through a pilot study which was conducted by taking a small sample size, including students from all professional years (n = 20). After getting a feedback from the students, necessary changes were made to the questionnaire with the aim of making it more brief and simple. The reliability coefficient of the questionnaire was calculated by using SPSS v.20. The Cronbach’s alpha value of 0.71 was computed. The responses of pilot study were not included in final analysis.

The questionnaire was formatted as a paper based survey and was divided into five sections. The first part was about socio-demographic characteristics of the students. This covered age, gender, family residential status like urban and rural background, year of study, family income status, family business, past entrepreneurship experience. The second part evaluates the behavioural belief of the students about pharmacy ownership. The third part includes statements on the control beliefs of students about pharmacy ownership. The fourth part assessed the normative beliefs of study participants’ students about pharmacy ownership. The fifth part of the questionnaire assessed the intent of entrepreneurship among participants. The total survey items comprised of 26 questions, eight questions related to demography of the respondents, six questions related to behavioural belief, seven questions related to control beliefs, four questions were related normative beliefs and one question about intent of entrepreneurship among study respondents.

To date, there is no ethical requirement for non-clinical observational studies in Pakistan [13, 14]. However, permission was taken from the respective course coordinators and lecturers of the school prior to data collection. Students were comprehensively explained about the objectives of this study before requesting them for their voluntary participation in this study. Written consent was taken from participants prior to data collection. Data was dealt with high level of anonymity and confidentiality.

Statistical analysis of data was carried out on SPSS v.20. Descriptive analysis was applied to present the results in frequency and percentage. Behavioural beliefs, control beliefs and normative beliefs were assessed on four point Likert scale: 1 = strongly disagree, 2 = disagree, 3 = agree, 4 = strongly agree. Scores were summed and then dichotomized based on an arbitrary 50 % cut-off score. Reverse coding was also used for negative worded questions. Attitude was defined as an organized set of feelings which influences an individual’s behaviour. Attitudes score ranged from 6–24 (based on six statements). A cut-off score of <15 was taken as negative attitude (disliking about pharmacy ownership) while score of ≥15 as positive attitude (liking about pharmacy ownership). Beliefs were defined as individual’s perception of societal norms, ease or difficulty of performing behavior, and consequences of particular behaviour. An arbitrary 50 % cut-off score was used to categorize positive and negative beliefs of the participants. Perceived behavioural control towards pharmacy ownership was measured on scale of 7–28 (based on seven statements). Score of <18 were taken as negative attitudes while score of ≥18 as positive attitudes. Likewise, participants’ response of subjective norms towards pharmacy ownership was measured on scale of 4–16 (based on four statements). Score of <10 were taken as negative beliefs while score of ≥10 as positive beliefs. Association of independent variables (demography, behavioural beliefs, control beliefs, normative beliefs) with dependent variable (pharmacy ownership intention) was examined by employing logistic regression analysis. A *p* value of less than 0.05 was taken as significant.

## Results

A total number of 313 students participated in this study giving a response rate of 89.4 %. Majority of them were females (n = 231, 73.8 %), aged <21 years (n = 175, 55.9 %), belonged to urban areas (n = 241, 77 %) and had a cGPA of ≥2.8 (n = 263, 84 %). The complete information about the participants is presented in Table 1.

**Table 1:** Demographic characteristics of participants

Demographic variables	N (%)
Age (in years)^a^	
<21	175 (55.9)
≥21	138 (44.1)
Gender	
Female	231 (73.8)
Male	82 (26.2)
Family residential status	
Rural	72 (23)
Urban	241(77)
GPA^b^	
<2.8	50 (16)
≥2.8	263 (84)
Professional year	
1	62 (19.8)
2	71 (22.7)
3	70 (22.3)
4	58 (18.5)
5	52 (16.7)
Family income status	
≤30,000	36 (11.5)
31,000–60,000	102 (32.6)
61,000–90,000	137 (43.8)
91,000–120,000	20 (6.4)
>120,000	18 (5.8)
Do you have any family business?	
Yes	83 (26.5)
No	230 (73.5)
Do you have any past experience of entrepreneurship?	
Yes	22 (7)
No	291 (93)

Grade B is awarded to student with cGPA of 2.8

^a^ Mean ± S.D: 20.4 ± 2.68

^b^ Mean ± S.D: 3.23 ± 0.36

Table 2 describes the attitudes of students towards pharmacy ownership. Almost similar number of students agreed or strongly agreed that pharmacy ownership would make them professionally (82.1 %) and financially (81.8 %) successful. Students were also of the opinion that pharmacy ownership would greatly improve their management and marketing skills (94.2 %), and their sense of responsibility as a pharmacist (77.3 %). However, students were concerned about the risk of being entrepreneur as 67.1 % agreed or strongly agreed that pharmacy ownership would put them at risk during economic crises. Similarly, 78.6 % participants responded that pharmacy ownership would also increase their tax liability. Overall, the participants’ attitudes were negative towards pharmacy ownership (Mean score: 13.90  ± 0.41).

**Table 2:** Behavioural beliefs of participants towards pharmacy ownership

Attitude questions	Participants’ response N (%)			
	Strongly disagree	Disagree	Agree	Strongly agree
Pharmacy ownership would make me professionally successful (implementing pharmaceutical care services etc.)	2 (0.6)	54 (17.3)	202 (64.5)	55 (17.6)
Pharmacy ownership would make me financially successful	3 (1)	54 (17.3)	208 (66.5)	48 (15.3)
Pharmacy ownership would greatly improve my management and marketing skills	1 (0.3)	17 (5.4)	221 (70.6)	74 (23.6)
Pharmacy ownership would greatly increase my sense of responsibility as pharmacist	9 (2.9)	62 (19.8)	169 (54)	73 (23.3)
Pharmacy ownership would put me at risk during economic crises	7 (2.2)	96 (30.7)	170 (54.3)	40 (12.8)
Pharmacy ownership would increase my tax liability	4 (1.3)	63 (20.1)	198 (63.3)	48 (15.3)

Attitude was assessed by giving 1 to SD, 2 to D, 3 to A, 4 to SA. The scale measured attitude from maximum 24 to minimum 6. Score of ≤15 were taken as negative attitude while >15 as positive attitude. Mean attitude score was 13.90  ± 0.41

The results showed that pharmacy students considered pharmacy ownership an extreme risk as they believed that not many people become successful, as more than half (56.2 %) of the participants agreed or strongly agreed to this point. Furthermore, remarkable number of students (91.4 %) believed that pharmacy ownership would require increase time commitments to fulfil professional responsibilities. Similarly, three-fourth of the students considered that their workload would significantly increase by owning a pharmacy. It is also noteworthy to highlight that 48.3 % students felt they do not have the knowledge and skills required to own a pharmacy. In general, participants’ response of perceived behavioural control towards pharmacy ownership was negative (Mean score: 16.88 ± 0.40) as summarized in Table 3.

**Table 3:** Control beliefs of participants towards pharmacy ownership

Questions	Participants’ response N (%)			
	Strongly agree	Agree	Disagree	Strongly disagree
Pharmacy ownership would not provide me a better quality of life	3 (1)	69 (22)	191 (61)	50 (16)
Pharmacy ownership would be extremely risk as not many people become successful	25 (8)	151 (48.2)	127 (40.6)	10 (3.2)
Pharmacy ownership would require increase time commitments to fulfil professional responsibilities	91 (29.1)	195 (62.3)	26 (8.3)	1 (0.3)
Pharmacy ownership would put additional burdens on me along with professional responsibilities	48 (15.3)	171 (54.6)	87 (27.8)	7 (2.2)
Pharmacy ownership would increase my work load	51 (16.3)	186 (59.4)	72 (23)	4 (1.3)
I would have to take a loan from the bank to own a pharmacy	78 (24.9)	198 (63.3)	36 (11.5)	1 (0.3)
I feel that I do not have the knowledge and skills to own my pharmacy	19 (6.1)	132 (42.2)	137 (43.8)	25 (8)

Control beliefs was assessed by giving 1 to SA, 2 to A, 3 to D, 4 to SD. The scale measured attitude from maximum 28 to minimum 7. Score of <18 were taken as negative attitudes while ≥18 as positive attitudes. Mean score of perceived behavioural control was 16.88 ± 0.40

Participants’ response of subjective norms towards pharmacy ownership is presented in Table 4. It was observed that students agreed or strongly agreed that if they own a pharmacy, they would be highly valued in their peer group (80.8 %) and society (60 %) respectively. Also, 67.7 % students believed that their family would want them own a pharmacy after they graduate. Participants’ overall response towards subjective norms questions was negative (Mean score: 9.66 ± 0.49).

**Table 4:** Normative beliefs of participants towards pharmacy ownership

Questions	Participants’ response N (%)			
	Strongly disagree	Disagree	Agree	Strongly agree
I would be highly valued in my peer group if I own a pharmacy	3 (1)	57 (18.2)	216 (69)	37 (11.8)
I would be highly valued in society if I own a pharmacy	6 (1.9)	119 (38)	165 (52.7)	23 (7.3)
My family thinks that I should own a pharmacy as soon as I graduate	9 (2.9)	92 (29.4)	181 (57.8)	31 (9.9)
My family thinks that I should own a pharmacy sometime in the future	34 (10.9)	199 (63.6)	63 (20.1)	17 (5.4)

Subjective norms were assessed by giving 1 to SD, 2 to D, 3 to A, 4 to SA. The scale measured attitude from maximum 16 to minimum 4. Score of <10 were taken as negative beliefs while ≥10 as positive belief. Mean score of subjective norms was 9.66 ± 0.49

It was observed that male students were less likely to own a pharmacy as compared to their female counterparts, however the association did not appear to be statistically significant (OR 0.75, p > 0.05). Similarly 3rd and 4th year students had more intent to own pharmacy in contrast to year one students (OR 3.29, p < 0.05; OR 7.36, p < 0.05 respectively). Surprisingly, students who had any family business were less likely to own a pharmacy (OR 0.34, p < 0.05). The results also showed that behavioural beliefs (OR 2.09, p < 0.05), control beliefs (OR 2.10, p < 0.05) and normative beliefs (OR 11.14, p < 0.05) were significantly associated with intention to own pharmacy as compared to their respective groups (Table 5).

**Table 5:** Association of demographic variables, attitudes and beliefs with the intent to become pharmacy owner

Variables	Intent to become entrepreneur (%)		Multivariate analysis	
	Yes	No	OR (CI)	p value
Gender				
Female	81.4	18.6	Ref	0.47
Male	72	28	0.75 (0.35–1.62)	
Professional year				
1	61.1	38.9	Ref	
2	71.8	28.2	1.44 (0.49–4.17)	0.50
3	87.9	12.1	3.29 (1.23–8.79)	0.014
4	84.3	15.7	7.36 (1.56–34.58)	<0.001
5	85.2	14.8	7.41 (1.48–18.95)	<0.001
Age (in years)				
<21	74.3	25.7	Ref	0.63
≥21	84.8	15.2	1.30 (0.44–3.80)	
cGPA				
<2.8	74	26	Ref	0.64
≥2.8	79.8	20.2	1.24 (0.49–3.12)	
Family residential status				
Rural	77.8	22.2	Ref	0.42
Urban	79.3	20.7	1.42 (0.59–3.38)	
Family income status				
≤30,000	77.4	22.6	Ref	
31,000–60,000	77.5	22.5	0.72 (0.32–1.61)	0.43
61,000–90,000	88.9	11.1	2.38 (0.60–9.46)	0.21
91,000–120,000	95	5	6.54 (0.68–62.50)	0.10
>120,000	61.1	38.9	1.29 (0.30–5.41)	0.72
Family business				
No	84.3	15.7	Ref	0.006
Yes	63.9	36.1	0.34 (0.15–0.73)	
Past entrepreneurship experience				
No	81.1	18.9	Ref	0.254
Yes	50	50	0.49 (0.14–1.6)	
Attitude				
Negative	74.2	25.8	Ref	0.029
Positive	85.8	14.2	2.09 (1.46–4.57)	
Perceived behavioural control				
Negative	65.6	34.4	Ref	0.038
Positive	81.1	11.9	2.10 (1.04–4.2)	
Subjective norms				
Negative	44.7	55.3	Ref	0.001
Positive	91.7	8.3	11.14 (5.6–21.1)	

Overall predictive accuracy of the model is 77.7 %

Omnibus tests of model coefficients: Chi square value = 213.795, p < 0.001

−2 Log Likelihood = 752.030, Nagelkerke R square = 0.274

*OR* odds ratio, *CI* confidence interval

## Discussion

The results suggest that participants’ attitudes towards pharmacy ownership were negative. These findings are not in accordance to a study where participants showed positive attitude towards pharmacy ownership [15]. A relatively small number of participants agreed that pharmacy ownership would make them professionally and financially successfully. Similarly, they also considered pharmacy ownership as a financial risk during economic crises. These findings reflect their negative attitudes and their lack of information about pharmacy ownership. These results could be related to another research where same results were reported [16]. Educating pharmacy students about the benefits of pharmacy ownership could aid in changing the attitudes of pharmacy students towards ownership. Additionally, their negative thoughts of ownership mainly from financial perspectives could also be addressed by developing entrepreneurial skills in pharmacy students through both didactic and experiential work in the form of entrepreneurial pharmacy practice program. Not many students agreed that pharmacy ownership would improve their management and marketing skills. Researchers revealed that marketing skills are one of the significant predictors of ownership intention [17]. Therefore, these results could be taken into consideration while enhancing the ownership spirits among pharmacy students.

In response to perceived behavioural control, almost half of the participants agreed that they do not have the knowledge and skills to own a pharmacy. The likely reason of these results could be due to lack of entrepreneurship content in pharmacy curriculum. There is a need to further explore the reasons which leads to these results, and curriculum should be revised accordingly. We also encourage the use of innovative learning approaches, like Problem Based Learning, to develop a range of ownership skills among students at pharmacy schools as supported by Refia and Thompson [18]. Fear of financial losses, lack of security, and work and time demands are some of the factors that discourage people from going into business [19]. The responses of pharmacy students were not very different as majority of them have the same concerns. There is a need to realize that not every pharmacy student can become entrepreneur as there are lot of challenges and insecurities that go with entrepreneurship [19]. It highlights the responsibility of academician and preceptors to play their role in identifying and subsequently nurturing students who have inclination towards entrepreneurship.

Although majority of the participants agreed to the subjective norms questions, overall beliefs of the respondents were negative. A large proportion of pharmacy students disagreed that they would be highly valued in society if they own a pharmacy. These results could be interpreted in two ways. Firstly, we need to look at the public awareness about the role of pharmacist in Pakistan. A study reported that patients’ opinions about the role of pharmacists are not optimized healthcare settings of Pakistan [20]. This amplifies the need to project more positive image of pharmacist and awareness on how they can be beneficial for the society. Secondly, it is pertinent to explore the attitudes of the public towards entrepreneurship and its importance in society as it is the key social and cultural norms. There is a need to promote culture of entrepreneurship in Pakistan, especially among pharmacy students because of many reasons. Firstly, this would help in strengthening community pharmacy setting in Pakistan as researchers have reported the scarcity of qualified pharmacist in majority of pharmacies in Pakistan [21]. Secondly, with the growing number of pharmacy students graduating every year, there is an extreme risk of exacerbation of unemployment [22]. In view of this, there is a pressing need to design effective interventions in the form educational programs for the public to raise their understanding of entrepreneurship, which in turn would encourage the pharmacist to take up this important role to meet the needs of the society.

This study also explored the factors associated with the intention of pharmacy students to become entrepreneur. Several studies reported that women are less likely than men to pursue a male dominated profession such as entrepreneurship [23, 24]. More studies are required to explore how gender affects entrepreneurial behaviour in Pakistani community. It was also observed that senior pharmacy students were more likely to have positive intent towards entrepreneurship. The likely reason of such results could be the increase exposure of senior pharmacy students to community pharmacy training, hospital clerkship and pharmacy management courses. Entrepreneurship curriculum should be developed in a way to expose students to the basics of entrepreneurship in early years of pharmacy education, followed by advanced levels in later years of pharmacy education. The study also highlights the influence of family business on the intentions of pharmacy students to pursue entrepreneurship. The findings of our study were contradictory to traditional entrepreneurship research [25]. We found that students having family businesses were less likely to own a pharmacy than the other group. Presumably, students born and raised in a business environment may have been affected by the drawbacks of entrepreneurship in the form of sacrifices and absence of parents due to business issues. A holistic view of entrepreneurship should be provided to pharmacy students by highlighting the benefits, along with the constraint associated with entrepreneurship.

This study furthers the theory of planned behaviour as the results show that attitudes, subjective norms and perceived behavioural control are the significant predictors of intention of pharmacy students to own a pharmacy. The positivity in the beliefs of the students has led them to a favourable intention to own pharmacy. These results are in line with several other studies that reported the same results [12, 26–28]. The findings of these results could become a basis to design effective entrepreneurship program for pharmacy students by adopting the theoretical considerations and the empirical findings of this study.

The strength of this study is that it has highlighted the area where not much work has been done in Pakistan, and around the world. This study was an attempt to fill the gap in the existing knowledge and make important contribution to pharmacy education and entrepreneurship literature. This study has focused on exploring the factors contributing to the intention of pharmacy students towards pharmacy ownership, which will contribute valuably to the existing literature. This study also paves the way for future research in different directions. It would be interesting to know the intentions of pharmacy students in other universities of Pakistan. This study would encourage the pharmacy researchers and academicians to develop, implement and assess entrepreneurship course by exploring the perception of pharmacy students. Future studies could also assess the behaviour of these students after their graduation.

Like any other research, this study is not free from limitations. The results of this single centre study could affect the generalizability of the results. Though this study considers normative influences, it still does not take into account environmental or economic factors that may influence students’ intention to own pharmacy. Moreover, the possibility of potential interactions between variables due to confounding factor, and the possibility of inherent bias may limit the validity of our findings.

## Conclusion

Overall, the behavioural beliefs, normative beliefs and control beliefs of the pharmacy students were negative towards pharmacy ownership. Professional year and family business were the significant predictors of the intentions of pharmacy students to own pharmacy. Implementation of entrepreneurship course in pharmacy school may transform the beliefs of participants towards pharmacy ownership.
